# Study of Waste Jute Fibre Panels (*Corchorus capsularis* L.) Agglomerated with Portland Cement and Starch

**DOI:** 10.3390/polym12030599

**Published:** 2020-03-06

**Authors:** Maria Teresa Ferrandez-García, Clara Eugenia Ferrandez-Garcia, Teresa Garcia-Ortuño, Antonio Ferrandez-Garcia, Manuel Ferrandez-Villena

**Affiliations:** Department of Engineering, Universidad Miguel Hernandez, 03300 Orihuela, Spain; mt.ferrandez@umh.es (M.T.F.-G.); cferrandez@umh.es (C.E.F.-G.); tgarcia@umh.es (T.G.-O.); antonio.ferrandezg@umh.es (A.F.-G.)

**Keywords:** cement-bonded, starch, waste jute, composite, physical, mechanical and thermal properties

## Abstract

This paper presents an experimental study on the bond behaviour of cement panels reinforced with plant fibres from the recycling of waste jute bags, using starch as a plasticiser. During processing, different proportions of jute (5 wt %, 10 wt %, 15 wt %, and 20 wt %) were used with respect to the weight of cement, and the mixture was exposed to a pressure of 2.6 MPa and a temperature of 100 °C. The density, swelling thickness, internal bonding, flexural strength, and thermal conductivity were studied. Mechanical tests indicated that the values of the modulus of rupture (MOR) and the modulus of elasticity (MOE) increased over time; thus, the jute particles appeared to be protected by the plasticised starch and no degradation was observed. At 28 days, the particleboard with 5% starch had an MOR of 12.82 MPa and an MOE of 3.43 GPa; these values decreased when the jute proportion was higher. The thermal conductivity varied from 0.068 to 0.085 W·m^−1^·K^−1^. The main conclusion is that jute-cement-starch composite panels can be manufactured with physical, mechanical, and thermal properties that meet the European standards for use in the construction of buildings as partitions, interior divisions, and thermal insulators.

## 1. Introduction

Industrial building construction uses large amounts of prefabricated panels for interior partitions. The installation of prefabricated panels is not only more economically profitable, but it also provides sustainable products that generate energy savings. Aerated concrete, polystyrene core composites, particleboard or plywood panels, and drywall or fibre-cement sheeting are currently used for interior partitions. Synthetic fibres are commonly used as the main reinforcement in cement-based composites. However, the production of synthetic fibres has the drawback of consuming large amounts of energy and chemical and petrochemical products. Consequently, new research trends focus on the alternative use of plant fibres as reinforcement. The use of plant fibres as reinforcement has been shown to improve the mechanical characteristics of cement, leading to the manufacture of materials that can be used in construction [1]. Similarly, the use of plant fibres from plant waste would contribute to recycling and recovery of this type of waste.

Concrete lightened with vegetable fibres is important from a commercial perspective owing to its low thermal conductivity and high acoustic absorption [2]; however, plant fibres generally present degradation problems with cement [3]. Some tests of fibre-cement composites have shown that, after one year of exposure in temperate or tropical environments [4], they underwent a severe decrease in flexural strength and modulus of elasticity in bending, most likely resulting from the carbonation of the cement matrix followed by progressive microcracking.

Yan et al. [5] maintain in their review of the use of plant fibres in cement composite materials that cellulosic fibres present highly variable mechanical properties and that the main reasons for the degradation of cellulose tissue are alkaline degradation (hydrolysis) and mineralisation of the fibres. These mechanisms produce changes in the chemical composition of the fibres and diminish their strength. Several constituents of the biomass, such as soluble sugars or low-molar-mass hemicelluloses, adversely affect the preparation and performance of concrete [6]; therefore, selecting biomass sources with a low content of these compounds would minimise these drawbacks. Although using plant waste is seen as an improvement, some fibres cannot withstand the high pH of the cement; therefore, additional studies are required regarding the effect of plant fibres on the reduction of cracks, the structure of pores of the cement matrix, and the permeability to water and chloride [7]. The final properties of cement composites with cellulosic fibres depend not only on the fibre and the constituents of the cement matrix, but also on the manufacturing process [8].

Jute is among the plant fibres most commonly used as reinforcement in concrete and cement mortars, with a density of 1460 kg/m^3^ and water absorption of 13% [9].

The longitudinal modulus of elasticity (MOE) for jute has been estimated to be seven times greater than the transverse MOE [10].

The considerable reduction in the strength of materials made from jute fibres applied to cement mortars has been explained by the influence of the alkalinity of the mortar on the fibres [11]. In these compounds, a delayed hydration of the cement grains occurs, which increases with the jute content in the cement matrix [12]. This behaviour is attributed to the insoluble sugars leached from the fibre surface in the highly alkaline environment of the cement, forming a layer in the cement grains and preventing their hydration. The hydroxyl groups of jute are involved in its natural degradation; thus, the addition of a simple acetate group to the fibre changes its properties. To improve the interface adhesion between non-polar matrices and hydrophilic fibres, a coupling agent such as an unsaturated polyester resin must be used. In cement mortars with jute, the emulsion of a polymer was found to increase the compressive strength and the modulus of rupture (MOR) or the flexural strength [13]. With the application of a polymer, an interpenetrating network structure between the polymer and cement matrices is created and the sugars leached from the jute fibres are reduced, favouring the hydration of the cement [14]. Tests have been conducted on the strength of alkalised jute fibres [15,16]. In tests with cement mortars reinforced with jute fibres and treated with alkali, it was concluded that they exhibited improved properties [17,18]. Alkaline treatments combined with polymers have also been proposed to modify the jute fibres, improving the properties of fibre-based cement mortars [14,19,20]. Other authors have proposed treating plant fibres with different plasticised starches [21]. After 180 days of ageing, in tests of reinforced concrete strengthened with jute fabrics, the jute fibres showed substantial signs of degradation, whereas when a plasticiser was used, the fibres were protected [22]. Research on cement mortars reinforced with jute mesh and lightweight aggregates [23] showed that the maximum flexural strength increased with the increasing jute volume. Studies have also been conducted on the impact fracture properties of cement composites reinforced with jute fibres [24], demonstrating that the energy absorbed decreased over time, which confirmed the degradation of the fibre.

The dispersion of fibres has been identified as an important factor that influences the properties of short-fibre composites. A good dispersion of fibres promotes a good interfacial bond, reducing the gaps and ensuring that the fibres are completely surrounded by the matrix [25]. The dispersion can be influenced by manufacturing parameters such as temperature and pressure [26].

With concrete and cement mortars, organic additives have also been used for decades for their specific properties [27]. These additives may be native biopolymers (starch) or substituted polymers (cellulose ethers). Starch-based lightweight concrete has been found to be more economical than concrete of a similar density that uses perlite as an aggregate [28]. Similarly, the concrete density has been shown to decrease during curing and drying, where the water is retained by the starch gel, and then migrates to the outside of the concrete to finally evaporate [29]. Polysaccharides added to cement can be classified as water reducing agents and setting retardants [30,31]. Thin sheets can be manufactured using cement and starch subjected to pressure and heat in a hotplate press for 1 h, resulting in good mechanical properties and plasticising the starch in the process [32].

For industrial-scale production of cement–wood materials that use cellulose pulp as the only reinforcing element, a binder is added. The materials are exposed to pressure and heat for days, followed by a curing period at room temperature. A process used in other studies to obtain bonded cement consists of pressing and curing under saturated air conditions at 25 °C for two days, followed by a thermal curing process inside a chamber at 45 °C and 90% relative humidity (RH) for five days [4]. Another manufacturing process of cement panels consists of applying heat in a press, although an increase in temperature (>6 °C) over a period of days has been found to result in a general degradation of the mechanical properties [33]. Starch has been used since ancient times to preserve fabrics, so it serves to protect the jute fibres.

The objective of this research was to obtain a jute-particle polymer with cement, using potato starch as a plasticizer, via a manufacturing process involving compression and heat. In addition to making use of waste jute fibres from the textile industry, the novelty of the work presented here lies in the fact that the board production process uses lower temperature and pressure parameters than those used in the manufacture of industrial boards. This can lead to a considerable energy saving with respect to conventional board manufacture. The sheets were evaluated by meeting the European standards [34] in order to verify whether they can be used as a building material, studying their physical, mechanical, and thermal properties and analysing the influence of jute addition in different proportions and its stability over time.

## 2. Materials and Methods

### 2.1. Materials

The materials used were textile residues of jute fibres (*Corchorus capsularis*) in different proportions, CEM II/B-LL 32.5 N Portland cement, potato starch (*Solanum tuberosum*), and water.

**Figure 1:**
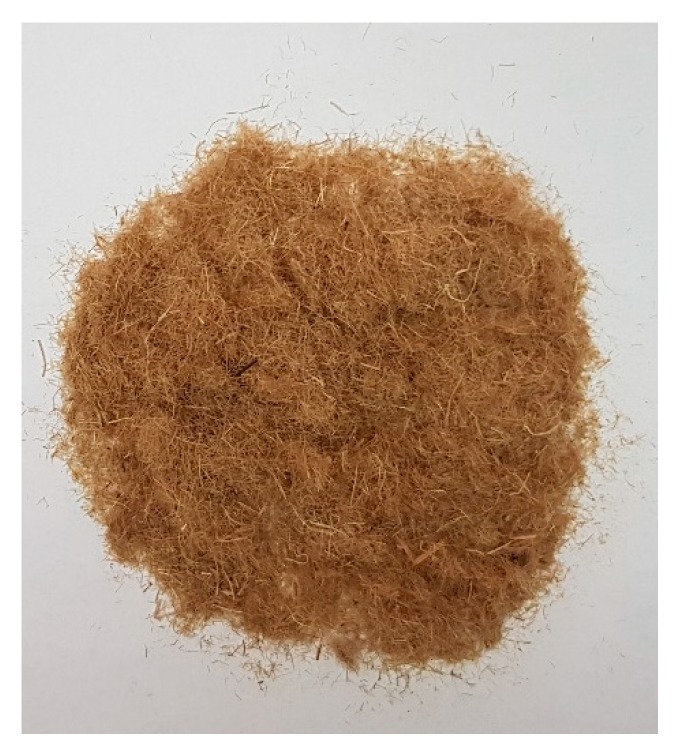
Jute particles used.

The jute particles (Figure 1) had a length of less than 5 mm and a diameter of 20 microns and were obtained from waste generated in a jute bag recycling facility. The aforementioned waste was destined for an authorised landfill. Potato starch of 90% purity from the food industry was used as a plasticiser. Chemically, starch is a mixture of two similar polysaccharides such as amylose and amylopectin. Potato starch typically contains large oval granules and gels at a temperature of 58–65 °C. Water was taken directly from the mains water supply, with an average temperature of 20 °C.

### 2.2. Manufacturing Process

The cement manufacturing process consisted of combining dry cement, starch, and jute particles that had been industrially processed for manufacturing sacks. The resulting raw meal was then pulverised with water at a pressure of 0.2 MPa, and the mixture was stirred for 10 min in an IMAL blender (model LGB100) at 30 rpm (IMAL S.R.L., Modena, Italy) until homogenised. The mixture was placed in a mould to obtain particleboards of dimensions 600 × 400 mm^2^, which were then pressed at 2.6 MPa and at a temperature of 100 °C for 1 h in a hotplate press. The particleboards were cooled in a horizontal position to room temperature. Afterwards, they were removed from the press and stacked in a horizontal position at room temperature for the first three days of setting, after which they were placed in a vertical position and kept under ambient conditions in the laboratory. Four types of panels with different dosages of jute particles were manufactured. According to the weight dosage, the percentage of each constituent in the mixture is shown in Table 1.

Twenty-eight days after manufacture, specimens were cut from four boards of each type using a circular saw. The other boards were cut 365 days after manufacture. In both cases, the specimens were previously kept for 24 h in a climate-controlled unit at a temperature of 20 °C and 65% humidity.

### 2.3. Methods

**Figure 2:**
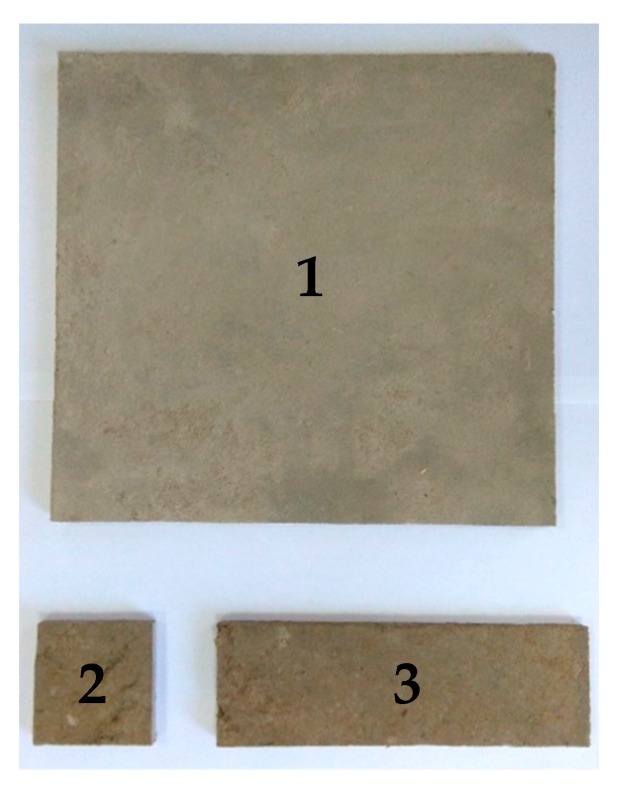
Specimens for the different tests. **1** (300 × 300 mm^2^), **2** (50 × 50 mm^2^), **3** (150 × 50 mm^2^).

The experimental tests were carried out in accordance with European standards applied to particleboards. The tests were carried out on four panels of each type 28 days after their manufacture and on another four after 365 days. The panels were cut with a circular saw to obtain specimens with the appropriate dimensions for each test. The specimens used in the tests are shown in Figure 2.

The physical properties evaluated were density [35], where six specimens of each particleboard with dimensions of 50 × 50 mm^2^ were used, and thickness swelling (TS) and water absorption (WA), where three specimens underwent tests after 2 and 24 h of immersion [36].

The mechanical properties evaluated were the MOR or flexural strength, modulus of elasticity in bending (MOE), and the internal bond or tensile strength (IB). The tests used to obtain the MOR and MOE were performed according to the European standard [37]. In the bending tests, three specimens of each particleboard with dimensions of 150 × 50 mm^2^ were used.

For the tests of IB, the European standard was applied [38]. The load was applied perpendicular to the specimen’s surface and at a constant speed during the entire test. For the perpendicular tensile tests, three specimens of each particleboard with dimensions of 50 × 50 mm^2^ were used.

The mechanical properties were determined using an IMAL IB700 universal testing machine (IMAL, S.R.L., Modena, Italy), which was operated at a speed of 5 mm min^−1^ for the bending test and 2 mm min^−1^ for the IB, as specified by the applicable standards.

Thermal conductivity was determined using the heat-flow-meter method [39]. Tests were performed for thermal conductivity on a heat-flow-meter instrument (NETZSCH Instruments North America, LLC., Burlington, MA, USA). One specimen for each particleboard with dimensions of 300 × 300 mm^2^ was used for this test. To compare the thermal properties of the particleboards, the thermal conductivity of the jute particles was also measured. This was done by pressing the particles with a pressure of 2.6 MPa to obtain mats. The thermal conductivity of panels made with cement and starch was also tested, using a weight % of 33% cement, 33% starch, and 33% water. In both cases, eight boards were obtained with approximate dimensions of 300 mm × 300 mm × 6 mm.

Table 2 shows the number of tests performed on each type of board to determine the different properties described above.

To check if the differences between the types of particleboards were significant, analysis of variance (ANOVA) of the mean values obtained for each panel and the standard deviation of each type of panel was performed. The differences between the types of particleboards were compared using the Duncan test (*p* < 0.05). For the statistical analysis, the program IBM SPSS Statistics v. 26.0 (IBM, Chicago, IL, USA) was used.

## 3. Results and Discussion

### 3.1. Physical Properties

The average values of the physical properties 28 days after manufacture are presented in Table 3. The sheet thickness increased according to jute content, varying from 6.2 cm in type A sheets to 6.9 cm in type D.

The density of the particleboards with jute fibres varied from 1321.8 kg m^3^ to 1405.2 kg m^3^, and it was found that the greater the proportion of jute fibres added, the lower the density.

The results for the specimens subjected to the TS test (after 2 and 24 h of immersion in water) indicate that the thickness swell increases as the wt % of jute in the mixture grows, although this is not proportional. No significant differences were found in the TS at 2 h and at 24 h of the panels with jute content types A, B, and C. According to the standards [39], sheets that are 6 to 12 mm thick with TS values of less than 14% after 24 h can be used in humid environments. Therefore, type A, B, and C panels with TS after 24 h between 8.8% and 9.9% could be used outdoors.

The percentage of water absorption (WA) increases proportionally with the percentage of jute particles in the dosage. Types C and D absorb half of the water during the first 2 h of immersion as after 24 h. Types A and B absorb one-third of the water after 2 h of WA as after 24 h. The WA of type A and B panels with jute particles after 24 h is 31%, a higher value than the 13% corresponding to the jute fibres [9]. This increase in WA can be caused by the porosity of the sheet. Type D boards have a large degree of thickness swelling and water absorption, which appears to indicate high porosity and suggests that the jute fibres are not fully coated; thus, the particles were not well dispersed.

The values obtained for TS after 24 h in this study are similar to those obtained in cement panels with 12% sisal, which exhibit a WA value slightly greater than 36% [4].

The average values of the physical properties obtained 365 days after the manufacture of the particleboards are presented in Table 4.

After one year, the density of the different types of boards does not vary significantly. In comparison with the density measured after 28 days, it can be observed that it has decreased in the boards that had the lowest proportion of jute and is similar in those with the greatest proportion. This may be because they have retained a greater proportion of water, subsequently helping to hydrate the cement.

The type A, B, and C particleboards have a TS of less than 10% after 28 days, whereas the type D particleboards reach a TS of 27%. After 365 days of curing, the TS decreases in the particleboards compared with the values obtained after 28 days, reaching a TS of 6.9% for the type B particleboards, while no significant differences were observed between the type A, B, and C particleboards. A similar behaviour can be observed with respect to WA throughout the curing time, obtaining the lowest value after 365 days for the type B particle boards, at 25.1%.

### 3.2. Mechanical Properties

**Figure 3:**
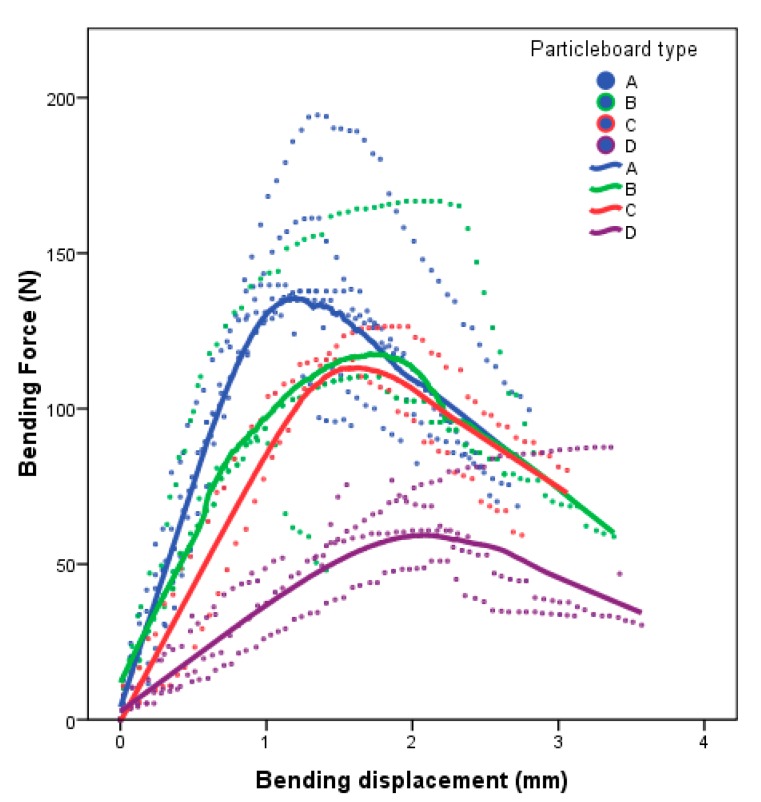
Average values of the bending tests for each type of particleboard.

The results of the bending test of the particleboards after 28 days of curing are shown in Figure 3.

The figure shows the fitted curves of the bending test for each type of panel; it can be observed that, in the elastic zone, the test fits a straight line. With a greater proportion of jute, the bending strength decreases and the maximum displacement increases; it is between 1.3 mm for 14.29 wt % of jute (type A) and 2.3 mm for 40 wt % of jute (type D). This indicates that, with a greater proportion of jute, the material becomes more deformable before reaching the breaking point.

**Figure 4:**
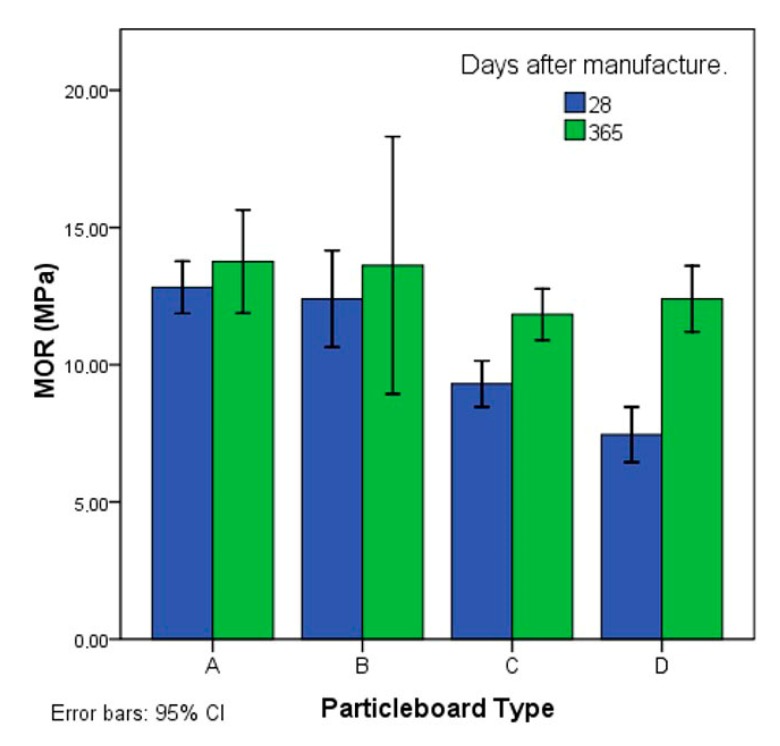
Modulus of rupture (MOR) after 28 and 365 days. CI, confidence interval.

**Figure 5:**
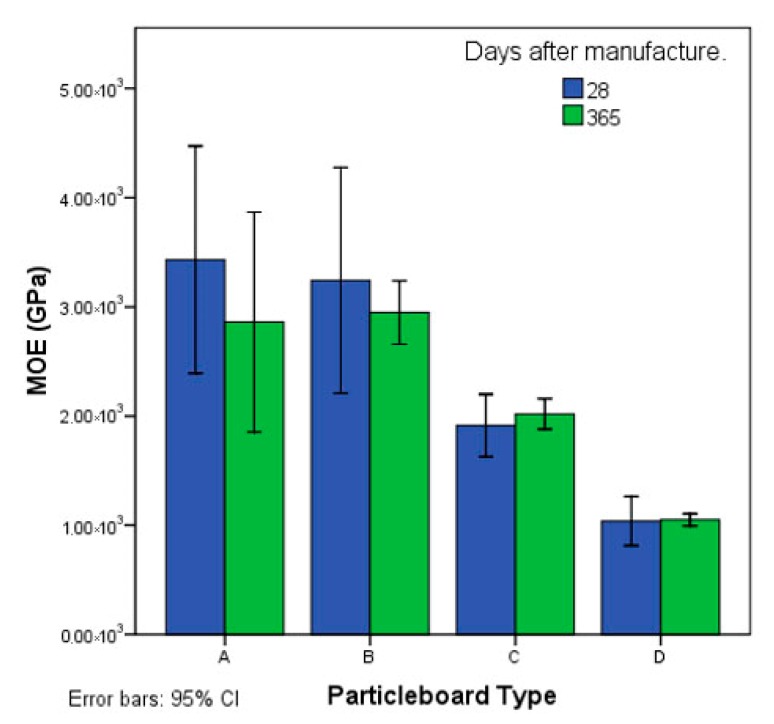
Modulus of elasticity (MOE) after 28 and 365 days. CI, confidence interval.

Figure 4 and Figure 5 show the average MOR and MOE values after 28 and 365 days of curing. In the tests after 28 days, the MOR values vary from 12.82 MPa for the type A particleboard to 7.45 MPa for the type D particleboard, which contains the highest proportion of jute particles. The MOR decreases with the increasing content of jute particles; therefore, it directly depends on the proportion of jute added. The type A and B particleboards are effective as type P2 particleboards and meet the European standards [34].

After 28 days of curing, the MOE of sheet type A is 3.43 GPa and decreases to 1.04 GPa in the particleboards that contain larger amounts of jute particles. Therefore, the greater the proportion of jute, the lower the MOE value, although the relationship is not linear. In the type A and B panels, the MOE values reach 3.43 and 3.24 GPa, respectively, which are higher than those required for type P2 particleboards [34].

To verify the possible degradation of particles and the strength of jute particles with cement using unmodified starch as a plasticiser, the bending test was carried out on particleboards after 365 days of curing at room temperature. The tests indicated that the flexural strength increased in the four types of particleboards, as shown in Figure 4; moreover, the increase in MOR was higher when the proportion of jute added was larger.

The MOE values were similar after 28 and 365 days (Figure 5). Degradation of the jute particles was not indicated in the bending tests; the particles were apparently protected from the alkalinity of the cement by the gelled starch.

**Figure 6:**
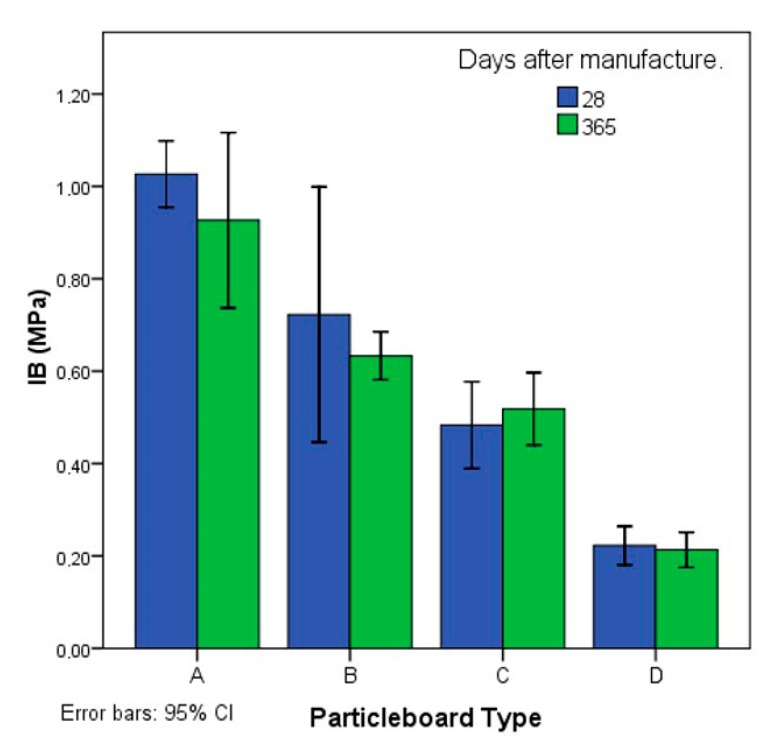
Average values of internal bond or tensile strength (IB) after 28 and 365 days of curing based on the type of particleboard. CI, confidence interval.

The average values in the IB tests after 28 and 365 days of curing are presented in Figure 6. The panels of types A, B, C, and D have IB values of 1.03, 0.77, 0.48, and 0.22 MPa, respectively. The decrease in the IB strength is proportional to the increase in jute particles.

In the IB tests after 365 days, the values of the type A and B particleboards decreased by approximately 11%, whereas the type C and D particleboards exhibited similar values. The curing time did not affect the internal tensile strength of the particleboards that contained more jute particles.

In general, if we compare the properties of the tested panels with those indicated in the specifications for particleboards [34], the type A and B particleboards can be classified as P2 class particleboards, which are used for interior and general applications. These are high-density particleboards and have higher MOE and IB values and a lower TS value than those required by the P2 classification.

Compared with other cement panels with plant fibres and other manufacturing processes, the panels in this study have lower density and greater flexural strength. The hydration of cement with starch and jute is slower and appears to be in accordance with that indicated in other studies [12,14,29,30,31]. In the literature related to jute with cement [5,12,13,14,15,16,17,18,19,20,24], the manufacturing process is different, and the studies are based on the degradation that occurs in the jute fibres when mixed with cement. In this study, recyclable jute particles from the industry were used, and the panels were manufactured by means of pressure and heat processes (100 °C for 1 h). Starch, which was used as a plasticiser, gelled in contact with jute during the process. All the results show that the gelled starch serves as protection for the jute particles, protecting the jute from degradation and favouring hydration of the cement, as observed with other plasticisers [21] and polymers [14].

### 3.3. Thermal Properties

The average results obtained for the thermal conductivity of each type of particleboard are reported in Table 5.

With the addition of jute, the thermal conductivity in the panels decreases. In the type C and D sheets with jute, no significant differences are observed, and the values are similar to those obtained with jute fibres without binder. Consequently, the manufactured panels are characterised by being good thermal insulators, emphasising that they have a lower density than the jute fibres. Cement-starch boards without jute particles have greater density and conductivity, and thus have worse properties as a thermal insulation material.

The thermal conductivity is inversely proportional to the voids in the composites. The packing of fibres into the matrix generates voids. Heat insulating properties of composites vary inversely to their density [40]; that is, heat insulating properties are enhanced when density decreases [41].

**Figure 7:**
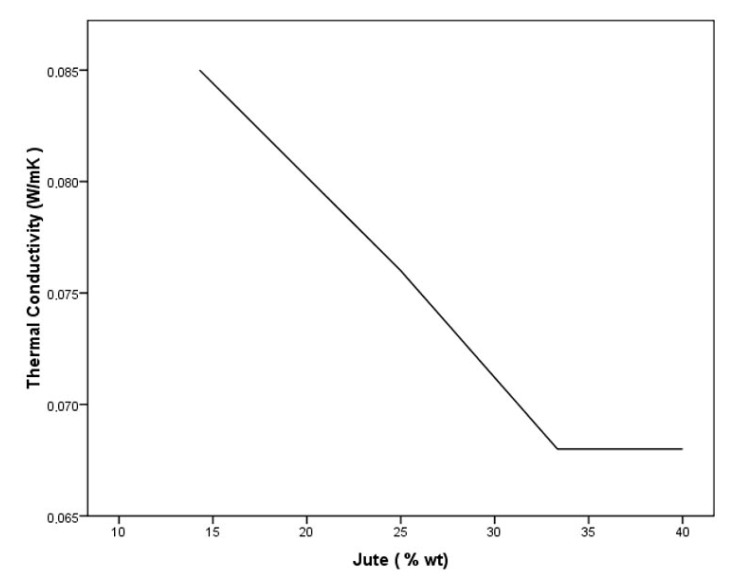
Thermal conductivity according to wt % of jute in the boards.

According to Table 5, the behaviour observed in this work is the same as that described by other authors. However, there is a point where the thermal conductivity remains almost constant, despite the fact that the density is lower (type C and D boards). We know that the density of the matrix (cement–jute) is greater than the density of jute fibres and that the porosity of the matrix increases with the addition of fibres. The decrease in thermal conductivity is related to the increase in porosity owing to the addition of particles [42]. With the addition of more than 33.34% wt of jute, it seems that the pores do not increase, and so the thermal conductivity does not increase, as can be observed in Figure 7.

To improve the thermal conductivity of the boards, it is necessary to analyse the ratio between the length and the diameter of the fibres, study the ideal water content, or add silica fume to optimise the matrix–jute interface.

Particleboards made of cement, sand, and coconut fibres [43] had a density of 1586.8 kg m^−3^ and a thermal conductivity of 0.651 W m^−1^ K^−1^. With the addition of 0.75% hemp fibres by volume to the concrete and a 10% reduction of coarse aggregates [44], a density of 2192.0 kg m^3^, an MOR of 3.1 MPa, and a thermal conductivity of 1.746 W m^−1^ K^−1^ were obtained. Therefore, the jute–cement–starch particleboards exhibit better thermal behaviour and lower density than those manufactured by other authors, which makes them effective as a thermal insulating material.

## 4. Conclusions

The results show that a manufacturing process using heat and pressure can produce composite panels of jute–cement–starch with physical, mechanical, and thermal properties that are compatible with use for interior cladding and furniture, reaching densities lower than those obtained with other plant fibres.

The use of recycled jute particles represents an alternative for achieving prefabricated panels made of agglomerated particles with cement and starch.

Composite panels with a proportion of 5 and 10 wt % of jute particles with respect to cement exhibit MOR, MOE, IB, and thermal conductivity values that make them suitable for thermal insulation in building construction, having properties for interior divisions, partitions, floors, dropped ceilings, and so on. Particleboards with a proportion of 15 or 20 wt % of jute could be used as thermal insulators.

The manufacture of jute–cement–starch panels consumes less energy than the currently manufactured industrial panels, as they are subjected to a pressure of 2.6 MPa and a temperature of 100 °C for 1 h, while the conventional wood–cement panels take 8 to 18 days to cure under a pressure of 3 and 3.5 MPa at temperatures of 75 to 80 °C.

The treatment that the jute fibres undergo before they are used in jute sacks (soaking and sulphonation) together with the addition of starch seems to influence the particles’ resistance to the alkalinity of the cement.

Gelled starch appears to protect the jute particles during the manufacturing process of the panels, similar to the function of other plasticiser products.

The use of industrial waste jute in the manufacturing of particleboards could have a positive application; its recycling contributes to the development of more sustainable materials, leading to an industrial and environmental benefit.

## Figures and Tables

**Table 1:** Types of panels manufactured.

Type of Particleboard	Number of Panels	Weight Dosage (%)			
		Jute	Cement	Starch	Water
A	8	14.29	28.57	28.57	28.57
B	8	25.00	25.00	25.00	25.00
C	8	33.34	22.22	22.22	22.22
D	8	40.00	20.00	20.00	20.00

**Table 2:** Number of tests performed on each type of board. TS, thickness swelling; WA, water adsorption; MOR, modulus of rupture; MOE, modulus of elasticity; IB, internal bond or tensile strength; TC, thermal conductivity.

Type of Test	Density	TS	WA	MOR	MOE	IB	TC
No. of tests performed on each type of board	48	24	24	48	48	24	8

**Table 3:** Physical properties after 28 days of curing.

Type of Particleboard	Thickness (mm)	Density (kg cm^3^)	TS 2 h (%)	TS 24 h (%)	WA 2 h (%)	WA 24 h (%)
A	6.19 (0.29)	1405.2 ^a^ (21.00)	4.2 ^a^ (0.30)	8.8 ^a^ (0.60)	12.1 ^a^ (1.00)	31.2 ^a^ (0.90)
B	6.34 (0.25)	1394.4 ^a^ (99.30)	3.2 ^a^ (0.10)	9.8 ^a^ (0.60)	12.2 ^a^ (0.30)	30.2 ^a^ (1.60)
C	6.78 (0.29)	1378.8 ^a^ (57.70)	5.7 ^a^ (2.00)	9.9 ^a^ (1.20)	29.9 ^b^ (9.80)	41.8 ^b^ (5.30)
D	6.88 (0.23)	1321.8 ^a^ (105.02)	17.8 ^b^ (3.40)	28.3 ^b^ (5,30)	55.2 ^c^ (11.80)	75.0 ^c^ (8.10)

( ) standard deviation. Duncan test (*p* < 0.05); ^a^, ^b^, and ^c^ indicate the same behaviours by these types of board, with no significant difference between them for the property analysed.

**Table 4:** Physical properties 365 days after the manufacture of the particleboards.

Type of Particleboard	Thickness (mm)	Density (kg cm^3^)	TS 2 h (%)	TS 24 h (%)	WA 2 h (%)	WA 24 (%)
A	6.13 (0.32)	1381.6 ^a^ (13.50)	1.4 ^a^ (0.20)	7.2 ^a^ (0.60)	11.4 ^a^ (1.30)	30.1 ^a^ (2.60)
B	6.27 (0.33)	1348.1 ^a^ (17.70)	1.2^a^ (0.10)	5.9 ^a^ (0.30)	10.0 ^a^ (0.10)	25.1 ^a^ (0.40)
C	6.65 (0.44)	1336.7 ^a^ (60.10)	1.7 ^a^ (0.60)	6.9 ^a^ (1.60)	12.8 ^a^ (0.90)	38.6 ^b^ (4.90)
D	6.81 (0.37)	1344.6 ^a^ (56.60)	7.3 ^b^ (0.60)	14.5 ^b^ (0.50)	43.7 ^b^ (3.10)	56.0 ^c^ (1.90)

( ) Standard deviation. Duncan test (*p* < 0.05); ^a^, ^b^, and ^c^ indicate the same behaviours by these types of board, with no significant difference between them for the property analysed.

**Table 5:** Thermal conductivity of the panels.

Type of Particleboard	Density (kg m^3^)	Thermal Conductivity (W m^−1^ K^−1^)	Standard Deviation (W m^−1^ K^−1^)
A	1405.6 ^a^	0.085 ^b^	0.009
B	1394.4 ^a^	0.076 ^ab^	0.005
C	1378.8 ^a^	0.068 ^a^	0.007
D	1321.8 ^a^	0.068 ^a^	0.006
Waste Jute	1460.0 ^b^	0.067 ^a^	0.005
Cement–starch boards	1578.9 ^c^	0.580 ^c^	0.036

Duncan test (*p* < 0.5); ^a^, ^b^, and ^c^ indicate the same behaviours by these types of board, with no significant differences between them for the property analysed.
